# Topology-aware validation of 3D skeleton kinematics for athletic movement classification using AthletePose3D

**DOI:** 10.3389/fphys.2026.1885404

**Published:** 2026-09-18

**Authors:** Hong Yang, Changdi Luo, Hewei Xiao

**Affiliations:** 1 School of Physical Education, Henan Normal University, Xinxiang, Henan, China; 2 Guangdong Preschool Normal College in Maoming, Maoming, Guangdong, China

**Keywords:** 3D human pose, AthletePose3D, athletic movement classification, graph convolutional networks, H36M-17, kinematic features, model attribution, random forest

## Abstract

**Background:**

Sports-specific three-dimensional pose data provide an opportunity to characterize the dynamics of athletic movement through joint trajectories and their temporal variation. This study investigated whether interpretable kinematic representations derived from 3D skeleton sequences can distinguish athletic movement classes and how their performance compares with topology-aware graph representations.

**Methods:**

AthletePose3D data from five subjects were analyzed using 18 movement labels derived from the available sequence annotations. H36M-17 pose sequences were root-centered and scale-normalized, and movement was represented using sequence-level features describing joint position, spatial range, velocity, acceleration, motion magnitude, and body-region relationships. These features characterize observable movement dynamics relevant to the study of coordinated human motion, but were not used to infer physiological mechanisms, injury risk, or causal biomechanics.

**Results:**

Interpretable kinematic representations consistently provided stronger movement-classification performance than the evaluated graph-based representations. The original class-balanced Random Forest achieved a mean macro-F1 of 0.854, and subsequent evaluation with a stronger tree-based classifier further improved performance, whereas fixed and adaptive graph approaches remained below the leading kinematic models. Model attribution indicated that discriminative information was distributed unevenly across body regions, with greater contributions from the lower limbs and selected upper-limb features, consistent with the multi-segment coordination required by the athletic movements represented in the dataset. Robustness and subject-domain-shift analyses further showed that classification performance depended on pose completeness and subject-action coverage.

**Conclusion:**

These findings demonstrate that normalized 3D skeletal kinematics can provide an interpretable representation of athletic movement dynamics for computational movement classification. The results support the use of pose-derived kinematic features for studying patterns of human movement while remaining distinct from direct physiological measurement or causal biomechanical inference.

## Introduction

Three-dimensional human pose analysis has become an increasingly important tool for studying movement because it converts complex visual motion into structured joint trajectories that can be analyzed computationally. In sports contexts, this capability is especially valuable because athletic performance depends on rapid, coordinated, and often high-acceleration movements that are difficult to characterize from video appearance alone. Recent progress in marker-less pose estimation, temporal pose modeling, and motion representation learning has expanded the possibility of using camera-based data for biomechanics and movement science; however, the reliability of these approaches depends strongly on whether the available datasets capture the movement conditions that the models are expected to analyze. General-purpose pose datasets and action-recognition benchmarks have supported major advances in human motion modeling, but they often contain everyday activities, staged motions, or relatively controlled movements rather than authentic sport-specific actions with abrupt acceleration, technical variation, and complex joint coordination (Pavllo et al., 2018; Li et al., 2021; Zheng et al., 2021).

Sports movement analysis presents a more demanding setting than many conventional human-pose tasks. Athletes frequently perform rapid changes in velocity, large joint excursions, rotational actions, jumps, throws, and contact-free but highly dynamic movement patterns. These characteristics create two related challenges. First, pose representations must preserve kinematic information that is relevant to movement class and technique. Second, downstream models must be evaluated with validation designs that account for subject, topology, camera, and class-support effects rather than relying on a single aggregate score. AthletePose3D was introduced to address part of this gap by providing a sports-specific 3D pose dataset for high-speed and high-acceleration athletic movements, with 12 broader sports-motion types and approximately 1.3 million frames. In the present study, the modeling label set consisted of 18 filename-parsed movement labels, including technique and error variants; these labels were used for classification analysis and should be understood as a finer modeling-level label set rather than as a replacement for the broader dataset-level sports-motion categories (Yeung et al., 2025).

Skeleton-based modeling provides a natural framework for this problem because the human body can be represented as a structured system of joints connected by anatomical relationships. Early temporal convolutional approaches demonstrated that 3D pose sequences contain strong temporal information for human motion estimation, while transformer-based pose models later showed the importance of jointly modeling spatial and temporal relationships among body joints (Pavllo et al., 2018; Li et al., 2021; Zheng et al., 2021). In parallel, skeleton-based action-recognition research has increasingly treated the skeleton as a graph, where nodes represent joints and edges represent anatomical or learned relationships. Spatial-temporal graph convolutional networks established this graph formulation as a central approach for skeleton-based action recognition, and later models extended it by incorporating multi-scale graph convolutions, action-specific links, and alternative skeleton representations such as 3D heatmap volumes (Yan et al., 2018; Li et al., 2019; Liu et al., 2020; Duan et al., 2022).

Despite these advances, graph-based skeleton modeling still requires careful benchmarking. A graph neural network may appear more anatomically appropriate than a tabular kinematic model, but this does not guarantee superior predictive performance in every dataset. Performance can depend on sample size, class support, temporal length, graph topology, preprocessing choices, and whether the graph structure preserves or suppresses discriminative joint-level variation. PoseC3D, for example, questioned whether graph sequences are always the most robust skeleton representation and showed that alternative 3D heatmap-based formulations can improve scalability and robustness in some action-recognition settings (Duan et al., 2022). Similarly, multi-scale graph methods such as MS-G3D emphasized that long-range and cross-spacetime dependencies matter, while actional-structural graph methods highlighted that fixed anatomical graphs may miss implicit action-specific joint relationships (Li et al., 2019; Liu et al., 2020). These findings suggest that skeleton topology should be evaluated empirically rather than assumed to be optimal.

For sports-biomechanics applications, interpretability is also essential. A movement-classification model may achieve high accuracy but still be difficult to trust if it does not reveal whether classification is driven by plausible regions of the body or by artifacts of data structure. Markerless motion-capture tools and pose-analysis pipelines have shown that camera-based movement analysis can provide useful kinematic information, but they also show that errors may arise from camera geometry, body scale, occlusion, and task-specific movement dynamics (Mathis et al., 2018; Karashchuk et al., 2021; Cotton et al., 2023; Jiang et al., 2023). Therefore, a robust sports-pose analysis pipeline should combine predictive benchmarking with dataset auditing, topology sensitivity, subject-level validation, and transparent model attribution. This is particularly important when movement labels include low-support technique or error subclasses, where aggregate accuracy may mask class-level instability.

The present study addresses these needs by evaluating normalized 3D H36M skeleton kinematics and topology-sensitive skeleton-graph representations for athletic movement classification using AthletePose3D. The analysis was deliberately framed as movement-classification and model-validation research, not as injury-risk prediction or causal biomechanical inference. The study had four aims. First, it audited the downloaded AthletePose3D files to establish analysis readiness, pose-array consistency, split structure, subject coverage, and H36M-17 skeleton topology. Second, it developed a strong baseline using interpretable normalized 3D kinematic features and repeated-seed classical classifiers. Third, it evaluated deep skeleton-graph models and graph-filtered topology controls to determine whether graph structure added predictive or validation value beyond the strongest kinematic baseline. Fourth, it summarized validation evidence and model attribution at joint and body-region levels to support transparent interpretation. This design was intended to provide an honest and reproducible evaluation of sports-pose movement classification, where graph topology is treated as a testable modeling assumption rather than as an automatic guarantee of superior performance.

## Methods

### Study design

This study was designed as a retrospective computational analysis of three-dimensional athletic movement data from AthletePose3D. The aim was to evaluate whether normalized 3D skeleton kinematics and skeleton-topology-sensitive graph representations could support athletic movement classification in a sports-specific pose dataset. The analysis was deliberately framed as movement-classification and model-validation research, not as injury-risk prediction, clinical diagnosis, or causal biomechanical inference. The full pipeline consisted of four stages: dataset-readiness auditing, kinematic baseline benchmarking, skeleton-graph/topology sensitivity analysis, and validation-oriented model attribution.

### Dataset

The study used AthletePose3D, a public sports-pose dataset developed for 3D human pose estimation and kinematic validation in athletic movements. The dataset was selected because it was specifically designed to capture high-speed and high-acceleration sports motions, which are underrepresented in conventional pose-estimation datasets. Only files used for non-commercial scientific analysis were processed. The raw download included the dataset archive, 3D pose archive, and camera-parameter file. The 2D pose archive was not required for the present analysis because the study focused on 3D skeleton-based movement classification.

### Data audit and analysis readiness

Before modeling, all downloaded and extracted files were indexed to verify dataset structure, split structure, subject coverage, action labels, file types, and 3D pose-array consistency. The audit recorded 48,017 indexed files, including 15,462 NumPy pose files, across three official dataset splits, five subjects, and 18 parsed athletic movement labels. Pose-array shape scanning confirmed that the H36M representation provided a consistent 17-joint, three-dimensional skeleton format. A camera-level analysis index and sequence-level analysis index were generated to prevent accidental mixing of file sources, duplicate representations, or unintended camera views. The H36M-17 anatomical skeleton was retained as the primary topology for graph-based analysis, with 17 joints and 16 anatomical edges.

### Pose representation and sample selection

The primary input representation was the H36M 3D pose sequence stored as NumPy arrays with shape T × J × 3, where T denotes the number of frames, J = 17 denotes the number of H36M skeleton joints, and the final dimension represents three-dimensional coordinates. To reduce multi-view duplicate inflation, the primary modeling dataset used camera 1 only. The final modeling set contained 635 camera-1 H36M pose samples distributed across 18 movement classes, with 374 training samples, 131 validation samples, and 130 test samples according to the official split. Additional camera-view and split-distribution outputs were retained for dataset-audit purposes, but they were not used to inflate the primary test set.

### Pose normalization

Each 3D pose sequence was normalized before feature extraction or graph modeling. For every frame, the root joint was subtracted from all joint coordinates to center the skeleton at the pelvis/root reference point. A robust scale factor was then computed as the median non-zero Euclidean distance of all joints from the root across the sequence. Coordinates were divided by this sequence-specific scale factor. If the computed scale was non-finite or non-positive, a scale value of 1.0 was used to avoid invalid transformations. This produced root-centered and scale-normalized skeleton sequences while preserving relative joint motion patterns.

### Kinematic feature extraction

For baseline classification, each normalized sequence was converted into interpretable sequence-level kinematic features. Features included frame count, joint count, per-joint coordinate means, coordinate standard deviations, coordinate ranges, velocity means, velocity standard deviations, mean absolute velocities, velocity-magnitude summaries, acceleration-magnitude summaries, global coordinate variability, global motion energy, global velocity, global acceleration, bounding-box ranges, bounding-box volume proxy, and left-right paired joint-distance summaries. This resulted in 387 kinematic features per sequence. These summary features were designed to provide a strong classical benchmark while preserving interpretability at joint and body-region levels.

### Baseline classifiers

Five baseline classifiers were evaluated on the kinematic feature matrix: class-balanced Logistic Regression, class-balanced Random Forest, HistGradientBoosting, class-weighted HistGradientBoosting, and a multilayer perceptron baseline. Missing values were imputed using the median estimated from the training set. Numeric features were standardized after imputation using training-set statistics only. The same preprocessing transformations were applied to validation and test data. Baseline models were evaluated using five random seeds: 11, 22, 33, 44, and 55. Class weighting was used where supported to reduce bias toward higher-support classes. The primary baseline selection criterion was mean macro-F1 on the official test set across repeated seeds, with balanced accuracy and weighted-F1 reported as supporting metrics.

### Skeleton topology construction

Skeleton topology analyses used the H36M-17 skeleton as the primary anatomical graph. The anatomical topology connected pelvis, hips, knees, ankles, spine, thorax, neck, head, shoulders, elbows, and wrists according to the H36M-style kinematic tree. To assess whether performance depended on the chosen topology, five graph-control settings were evaluated: anatomical H36M topology, symmetry-augmented topology, distance-based topology, fully connected topology, and random-matched topology. The symmetry-augmented topology added left-right paired joint edges. The distance-based topology was derived from average training-set joint geometry. The random-matched topology used the same approximate edge count as the anatomical graph but randomized the connected joint pairs. The fully connected topology acted as a dense graph control. Adjacency matrices were symmetrically normalized using degree normalization before graph filtering or graph-model input.

### Deep graph models

Deep skeleton-graph models were evaluated to test whether graph-based temporal modeling provided additional value beyond the classical kinematic baseline. Two graph architectures were implemented: a graph convolutional network and a graph-attention-style network. Input sequences were root-centered, scale-normalized, and temporally resampled to 64 frames. Each model used 17 skeleton joints with three coordinate channels as node features. Graph layers were followed by temporal convolution and sequence-level classification layers. Models were trained using the official training split, monitored using the validation split, and evaluated on the held-out test split. Training used class-weighted cross-entropy, AdamW optimization, early stopping based on validation macro-F1, batch size 32, learning rate 1 × 10^-^³, weight decay 1 × 10^-4^, dropout 0.25, hidden dimension 64, a maximum of 140 epochs, and early-stopping patience of 22 epochs. Models were repeated across the same five random seeds and evaluated across topology-control settings.

### Fast topology-filter sensitivity analysis

Because deep graph training is computationally expensive, a complementary fast topology-filter sensitivity analysis was also performed. In this analysis, normalized 3D pose sequences were graph-filtered using each normalized adjacency matrix before extracting the same kinematic feature set used in the baseline analysis. The identity/no-graph setting served as an unfiltered control, while anatomical, symmetry-augmented, distance-based, fully connected, and random-matched graph filters tested the effect of topology-induced smoothing. Random Forest and Logistic Regression classifiers were then trained on each graph-filtered feature representation using the same official split and repeated-seed design. This analysis was used as a topology-sensitivity benchmark and was not interpreted as a substitute for full deep graph training.

### Validation analyses

Validation was performed at multiple levels. First, all baseline and graph models were evaluated using the official train/validation/test split. Second, repeated-seed evaluation was used to quantify model stability. Third, topology sensitivity was assessed using anatomical, symmetry-augmented, distance-based, fully connected, and random-matched graph controls. Fourth, subject-holdout sensitivity was performed using the normalized kinematic features described above. For each held-out subject, the model was trained on all remaining subjects and tested on the held-out subject using only classes shared between training and test data. Subject S4 was excluded from completed subject-holdout metric reporting because it had insufficient common class coverage for a valid multi-class evaluation. Subject-holdout validation was therefore interpreted as a sensitivity analysis rather than a replacement for the official split.

### Model attribution

Model attribution was performed using the strongest kinematic baseline, the class-balanced Random Forest. A Random Forest model was refit on the official training split using the normalized kinematic features. Feature importance values were extracted from the fitted model and aggregated at three levels: individual features, H36M joint indices, and anatomical body regions. Feature names containing joint identifiers were mapped to the corresponding H36M-17 joint. Joints were further grouped into trunk, head-neck, left lower limb, right lower limb, left upper limb, right upper limb, and global feature categories. Attribution was reported as model-derived feature importance only and was not interpreted as causal evidence of biomechanical mechanisms.

### Performance metrics

The primary performance metric was macro-F1 because the task involved multiple movement classes with unequal class support, including low-support error subclasses. Balanced accuracy was reported to quantify average class-level recall. Additional reported metrics included accuracy, weighted-F1, macro precision, macro recall, macro receiver operating characteristic area under the curve, and average precision where probability outputs were available. Confusion matrices and class-wise precision, recall, F1-score, and support were retained for detailed reliability assessment. All metrics were calculated on held-out data only, with preprocessing parameters estimated from training data.

### Anatomically and motion-informed topology filters

To evaluate topology design more comprehensively, anatomically informed and motion-informed topology filters were introduced beyond the original anatomical, random-matched, and fully connected controls. These included a bone-length-weighted anatomical topology, joint-velocity-correlation topology, joint-acceleration-correlation topology, and a hybrid anatomy-motion topology combining anatomical structure with motion-derived dependencies estimated exclusively from the training split.

### Stronger tree-based comparator

A stronger tree-based comparator, ExtraTrees-Balanced, was added to evaluate whether the previous Random Forest benchmark underestimated the strength of classical kinematic models. This allowed for a more rigorous comparison between graph-based and non-graph-based approaches.

### Pose-perturbation stress testing

A pose-perturbation stress test was conducted to approximate downstream sensitivity to imperfect skeleton extraction. Perturbation conditions included Gaussian coordinate noise, random frame dropping, random joint masking, and a combined noise-mask-frame condition. These perturbations were applied only to the held-out test split, while training remained based on clean normalized pose features.

### Subject-domain-shift analysis

Subject-domain-shift analysis was introduced using a subject-action coverage matrix and common-label subject-holdout evaluation. This analysis directly quantified class-overlap instability rather than treating incomplete subject-holdout evaluation as a minor issue.

### Adaptive temporal graph model evaluation

Finally, a compact adaptive temporal graph model was evaluated using anatomical, anatomically informed, motion-informed, and hybrid topology initializations to test whether learnable topology refinement could close the performance gap with strong kinematic baselines.

### Software and reproducibility

All analyses were implemented in Python 3.12 using NumPy, pandas, scikit-learn, Matplotlib, and PyTorch. Dataset files were indexed without copying raw arrays into duplicated large directories. Intermediate outputs were saved as CSV files, Excel workbooks, Markdown reports, and high-resolution PNG figures. Random seeds were fixed for repeated-seed model evaluation. The final output package excluded raw dataset files and included analysis-ready tables, performance summaries, validation reports, figure files, and model-attribution outputs. The analysis was structured to preserve transparency, avoid data leakage from test data into preprocessing, and maintain a clear separation between dataset auditing, model training, validation, topology sensitivity, and attribution.

## Results

### Dataset readiness

The analysis-ready AthletePose3D dataset comprised 48,017 indexed files, including 15,462 NumPy pose files, across three official splits, five subjects, and 18 parsed athletic movement labels. The extracted H36M representation provided a consistent 17-joint, three-dimensional pose structure suitable for movement-classification analysis and skeleton-topology evaluation. **Figure 1** summarizes the extracted file composition, storage footprint, split-level sequence distribution, and subject-level sequence distribution, while **Table 1** provides the corresponding dataset-readiness audit and analysis-index outputs. The dataset was therefore retained for all downstream kinematic benchmarking, topology sensitivity, and attribution analyses.

**Figure 1:**
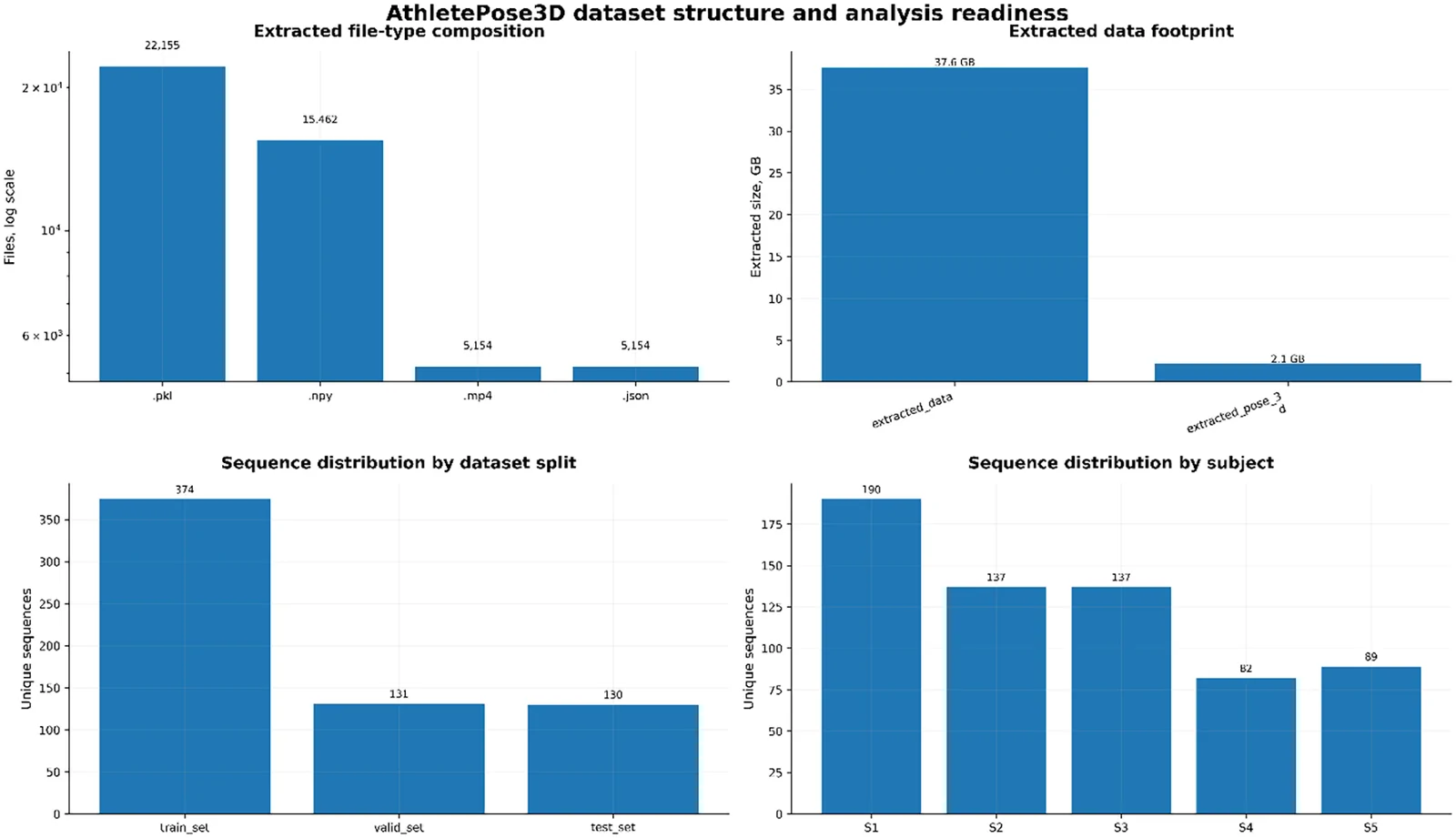
AthletePose3D dataset structure and analysis readiness. The figure summarizes extracted file-type composition, extracted data footprint, sequence distribution across official dataset splits, and subject-level sequence distribution used to verify readiness for 3D pose-based movement classification.

**Table 1:** Dataset-readiness audit for AthletePose3D.

Dataset-readiness item	Value
Indexed files	48,017
NPY pose files	15,462
Subjects	5
Parsed movement labels	18
Official splits	train_set, valid_set, test_set
Skeleton topology	H36M-17; 17 joints and 16 anatomical edges

The table reports file-type summaries, action distributions, subject distributions, sequence-level indexing, camera-level indexing, pose-array shape scans, sampled array-value profiles, and the H36M-17 skeleton topology used for downstream analysis.

### Baseline performance

The normalized 3D kinematic baseline produced strong movement-classification performance across the official train/validation/test split. The modeling dataset contained 635 camera-1 H36M pose samples distributed across 18 movement classes, with 374 training samples, 131 validation samples, and 130 test samples. Among the evaluated baseline models, the class-balanced Random Forest achieved the strongest repeated-seed performance, with mean macro-F1 of 0.854, balanced accuracy of 0.844, macro ROC-AUC of 0.992, and average precision of 0.937. **Figure 2** presents the full baseline performance dashboard, including macro-F1, balanced accuracy, metric profiles, and runtime comparison, while **Table 2** reports the complete repeated-seed baseline summary.

**Figure 2:**
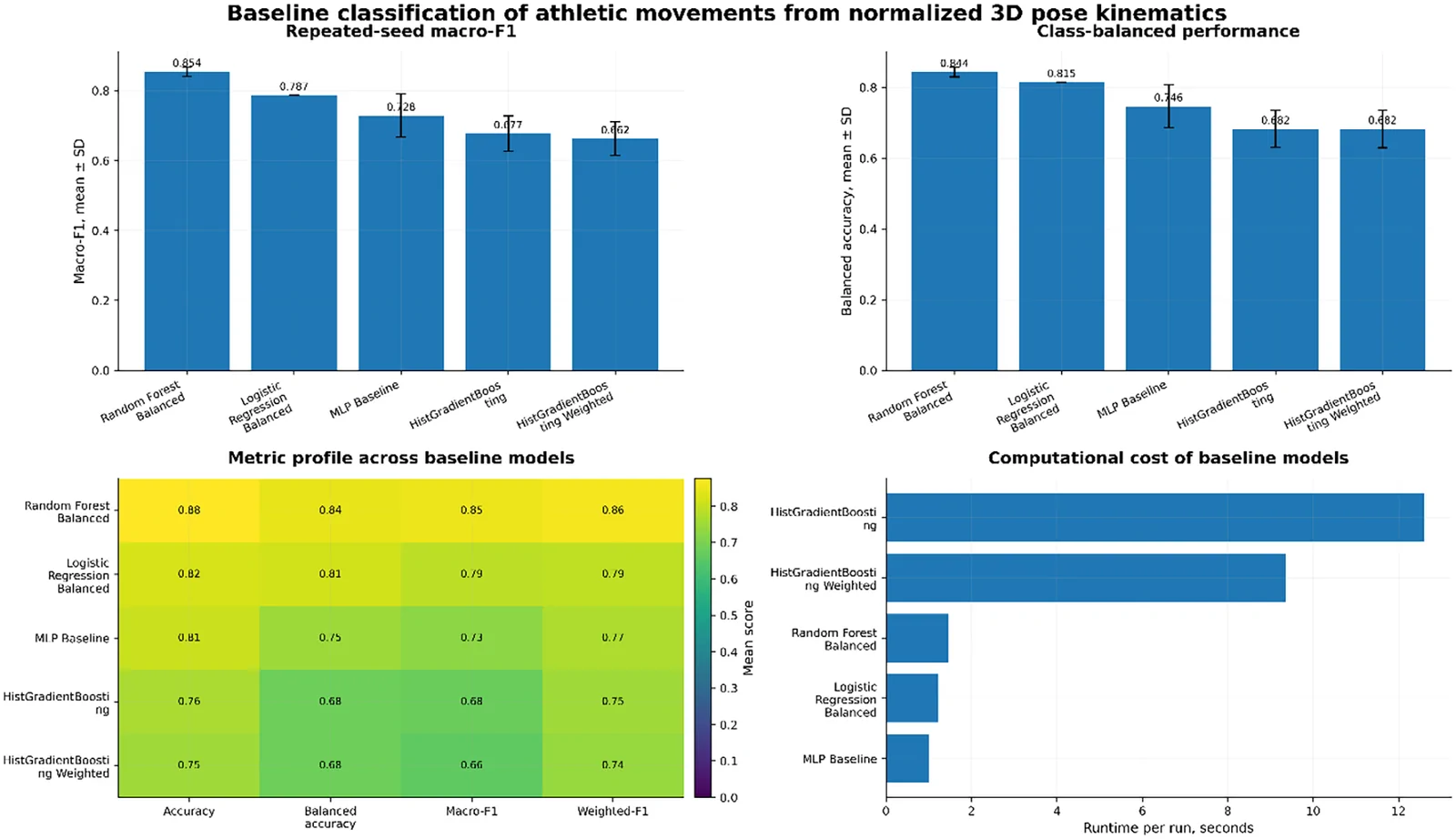
Baseline classification performance for normalized 3D pose kinematics. The figure reports repeated-seed macro-F1, balanced accuracy, multi-metric performance profiles, and computational runtime for the evaluated baseline classifiers.

**Table 2:** Repeated-seed baseline classification performance.

Model	Runs	Accuracy	Balanced accuracy	Macro-F1	Weighted-F1	Macro ROC-AUC	Average precision
Random Forest Balanced	5	0.875 ± 0.017	0.844 ± 0.014	0.854 ± 0.013	0.865 ± 0.019	0.992 ± 0.003	0.937 ± 0.016
Logistic Regression Balanced	5	0.823 ± 0.000	0.815 ± 0.000	0.787 ± 0.000	0.793 ± 0.000	0.984 ± 0.000	0.869 ± 0.000
MLP Baseline	5	0.806 ± 0.017	0.746 ± 0.060	0.728 ± 0.062	0.768 ± 0.025	0.980 ± 0.006	0.851 ± 0.017
HistGradientBoosting	5	0.765 ± 0.028	0.682 ± 0.052	0.677 ± 0.051	0.750 ± 0.030	0.976 ± 0.007	0.828 ± 0.029
HistGradientBoosting Weighted	5	0.751 ± 0.035	0.682 ± 0.053	0.662 ± 0.048	0.740 ± 0.032	0.969 ± 0.006	0.792 ± 0.045

The table reports accuracy, balanced accuracy, macro-F1, weighted-F1, macro precision, macro recall, macro ROC-AUC, average precision, and runtime across baseline classifiers.

### Validation evidence

The validation evidence showed that the original kinematic baseline remained stronger than the implemented fixed graph models, while graph and topology analyses provided additional validation layers (**Figure 3**). The class-balanced Random Forest baseline achieved macro-F1 of 0.854 and balanced accuracy of 0.844, the best fixed deep graph configuration achieved macro-F1 of 0.752 and balanced accuracy of 0.774, and the best fast topology-filtered configuration achieved macro-F1 of 0.852 and balanced accuracy of 0.844. Subject-holdout sensitivity showed heterogeneous subject-dependent performance rather than uniform cross-subject robustness. In the completed folds reported in **Table 3**, macro-F1 was 0.600 for S1, 0.895 for S2, 0.938 for S3, and 0.874 for S5, while S4 was excluded from complete multi-class reporting because only one common class was available. These results indicate that the subject-level analysis should be interpreted as a domain-shift sensitivity check, not as definitive evidence of broad subject-level generalizability.

**Table 3:** Subject-holdout sensitivity validation.

Held-out subject	Status	Train n	Test n	Classes	Accuracy	Balanced accuracy	Macro-F1	Weighted-F1
S1	complete	63	190	7	0.621	0.600	0.600	0.619
S2	complete	349	137	8	0.949	0.912	0.895	0.946
S3	complete	245	137	11	0.971	0.920	0.938	0.970
S4	Skipped insufficient common_classes	151	82	1	—	—	—	—
S5	complete	60	89	10	0.888	0.872	0.874	0.890

**Figure 3:**
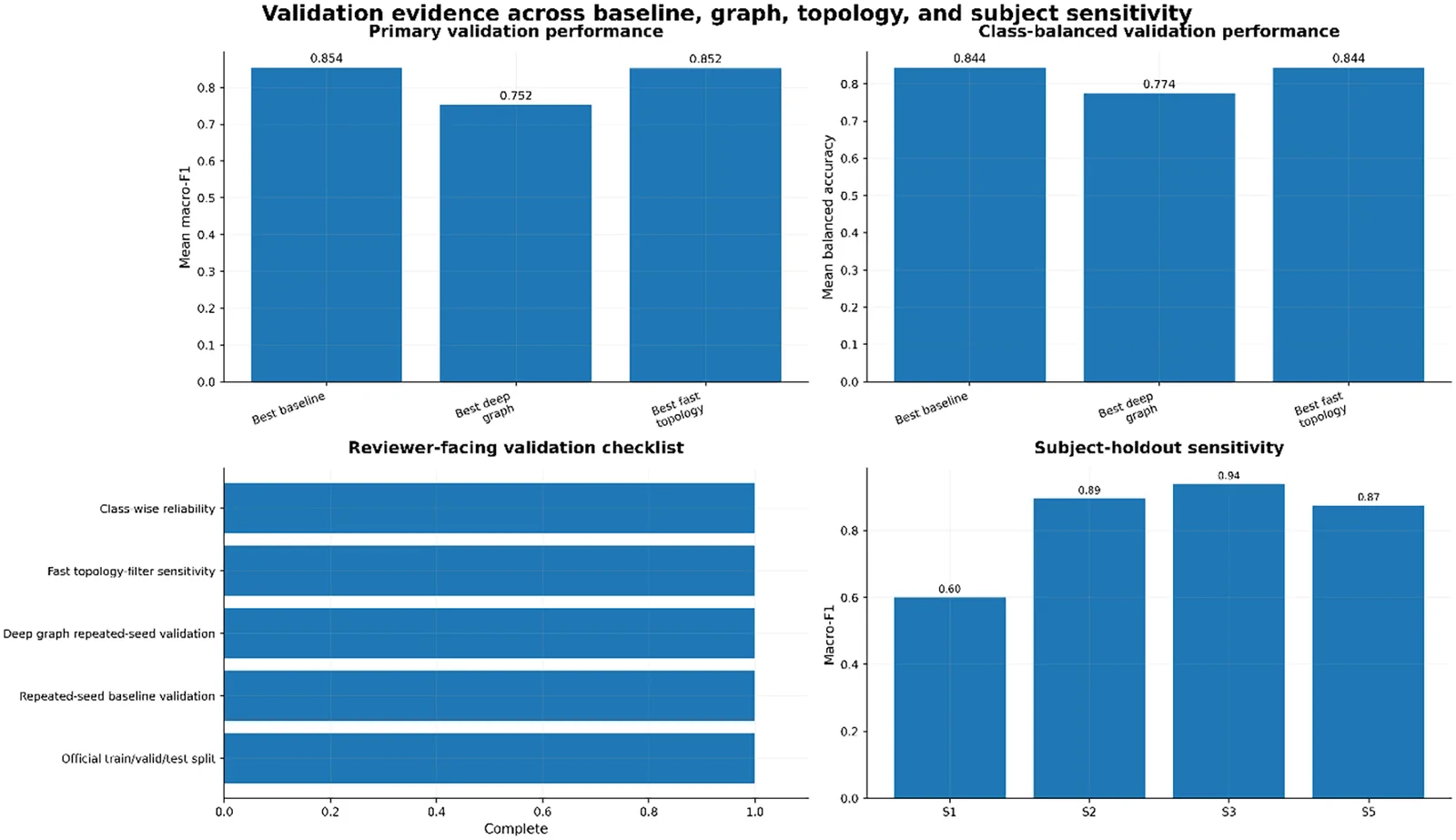
Validation evidence across baseline, graph, topology, and subject sensitivity analyses. The figure compares primary macro-F1, class-balanced accuracy, validation completeness, and exploratory subject-holdout macro-F1 across completed subject-level validation folds.

### Topology sensitivity

Topology-filtered sensitivity analysis showed that graph filtering did not improve over the strongest unfiltered kinematic baseline, but it clearly quantified the effect of different skeleton-topology controls. The identity/no-graph Random Forest configuration was closest to the normalized kinematic baseline, with macro-F1 of approximately 0.852 and a difference of −0.002 relative to the strongest baseline. Random Forest with anatomical H36M filtering was lower, with a macro-F1 difference of −0.056, while fully connected topology produced the largest degradation, with macro-F1 differences of −0.336 for Random Forest and −0.409 for Logistic Regression. **Figure 4** presents the topology-filtered models relative to the strongest baseline and the corresponding macro-F1 differences, while **Table 4** reports the complete topology-filtered performance comparison.

**Figure 4:**
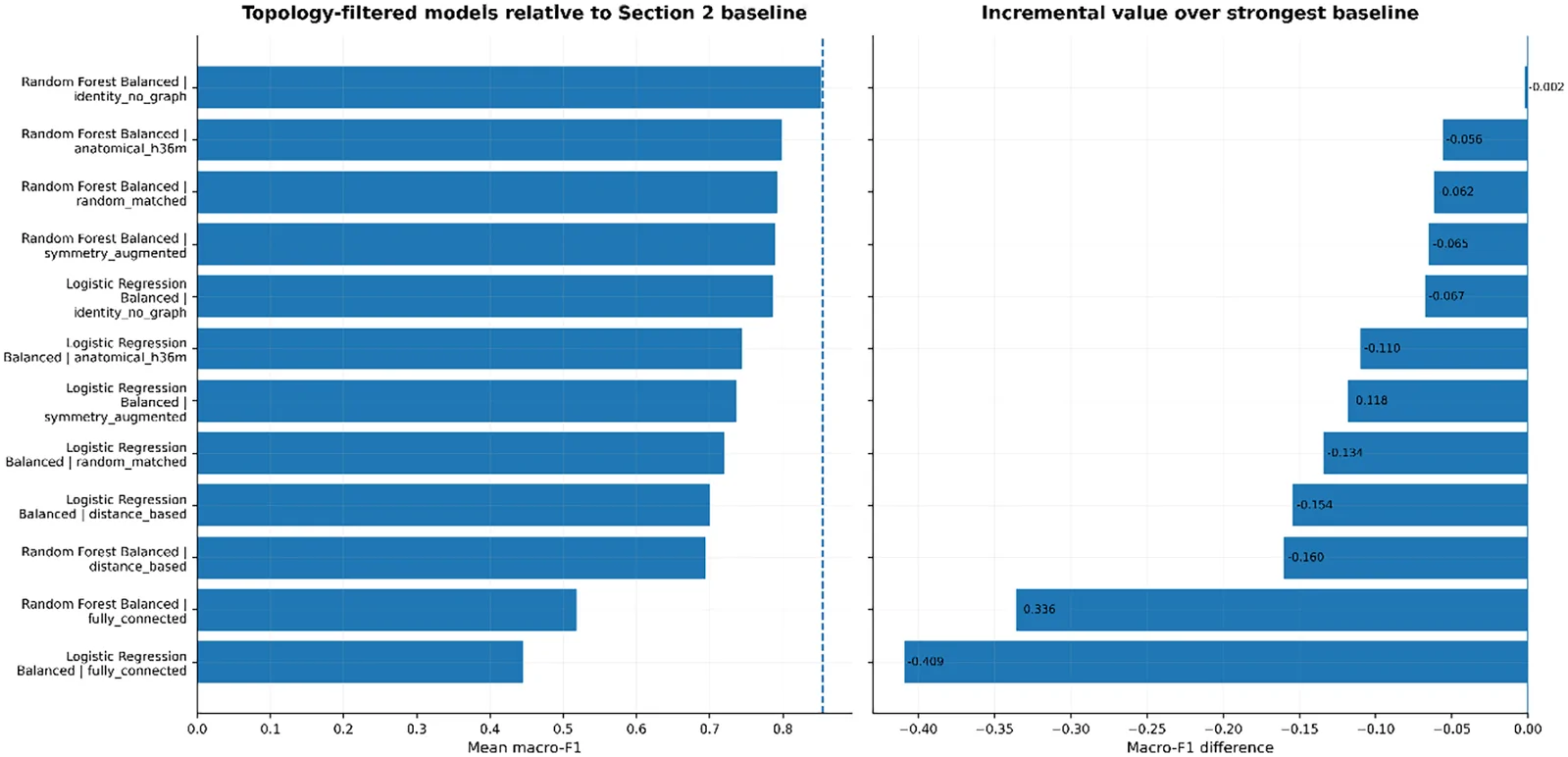
Graph-filtered topology controls relative to the strongest kinematic baseline. The figure compares topology-filtered classifiers against the normalized kinematic baseline and reports the incremental macro-F1 difference for each model-topology combination.

**Table 4:** Fast graph-filtered topology sensitivity relative to the strongest kinematic baseline.

Model	Topology	Macro-F1	Balanced accuracy	Weighted-F1	Baseline macro-F1	Δ Macro-F1	Δ Balanced accuracy
Random Forest Balanced	identity_no_graph	0.852	0.844	0.865	0.854	-0.002	0.000
Random Forest Balanced	anatomical_h36m	0.798	0.784	0.818	0.854	-0.056	-0.060
Random Forest Balanced	random_matched	0.793	0.797	0.830	0.854	-0.062	-0.046
Random Forest Balanced	symmetry_augmented	0.789	0.778	0.820	0.854	-0.065	-0.066
Logistic Regression Balanced	identity_no_graph	0.787	0.815	0.793	0.854	-0.067	-0.029
Logistic Regression Balanced	anatomical_h36m	0.744	0.765	0.787	0.854	-0.110	-0.079
Logistic Regression Balanced	symmetry_augmented	0.736	0.753	0.775	0.854	-0.118	-0.090
Logistic Regression Balanced	random_matched	0.720	0.740	0.763	0.854	-0.134	-0.104
Logistic Regression Balanced	distance_based	0.700	0.741	0.748	0.854	-0.154	-0.103
Random Forest Balanced	distance_based	0.694	0.706	0.690	0.854	-0.160	-0.137
Random Forest Balanced	fully_connected	0.518	0.549	0.586	0.854	-0.336	-0.294
Logistic Regression Balanced	fully_connected	0.445	0.474	0.548	0.854	-0.409	-0.370

### Deep graph models

Deep skeleton-graph experiments showed that model performance was sensitive to the choice of graph topology. The best repeated-seed deep graph configuration was the graph convolutional network using the anatomical H36M topology, which achieved mean macro-F1 of 0.752 and balanced accuracy of 0.774 on the official test split. This result did not exceed the strongest normalized kinematic baseline reported in **Table 2**, but it established that topology selection materially influenced graph-model performance. In particular, anatomically structured graph learning performed more reliably than arbitrary dense graph controls, supporting the inclusion of topology sensitivity as a validation component rather than treating graph construction as a fixed modeling assumption. The full repeated-seed GCN and GAT topology results are reported in **Table 5**.

**Table 5:** Deep graph topology performance summary.

Model	GCN	GAT	GAT	GCN	GCN	GAT	GAT	GCN	GCN	GAT
topology	anatomical_h36m	distance_based	random_matched	symmetry_augmented	distance_based	anatomical_h36m	symmetry_augmented	random_matched	fully_connected	fully_connected
n_runs	5	5	5	5	5	5	5	5	5	5
accuracy_mean	0.815385	0.801538	0.818462	0.78	0.764615	0.795385	0.8	0.8	0.627692	0.530769
accuracy_sd	0.045832	0.040118	0.048529	0.041281	0.06216	0.017709	0.057306	0.033086	0.026424	0.132567
balanced_accuracy_mean	0.774029	0.762037	0.758148	0.766537	0.7598	0.749259	0.750325	0.74049	0.64496	0.586366
balanced_accuracy_sd	0.043472	0.049248	0.04483	0.038466	0.048567	0.016813	0.059929	0.022678	0.044701	0.05638
macro_f1_mean	0.752429	0.734288	0.731703	0.730781	0.730507	0.719687	0.717605	0.710086	0.61487	0.506924
macro_f1_sd	0.060598	0.058368	0.054827	0.04927	0.052949	0.014582	0.066833	0.028078	0.055023	0.081283
weighted_f1_mean	0.780876	0.759359	0.786387	0.747446	0.737612	0.756468	0.75753	0.761506	0.605274	0.477844
weighted_f1_sd	0.056588	0.04585	0.055066	0.037279	0.047469	0.024072	0.061422	0.042165	0.020164	0.139989
macro_precision_mean	0.779993	0.745266	0.763251	0.745633	0.766949	0.7485	0.725392	0.73806	0.649467	0.515229
macro_precision_sd	0.084544	0.075602	0.070328	0.061221	0.048337	0.037694	0.067497	0.044115	0.06017	0.096725
macro_recall_mean	0.774029	0.762037	0.758148	0.766537	0.7598	0.749259	0.750325	0.74049	0.64496	0.586366
macro_recall_sd	0.043472	0.049248	0.04483	0.038466	0.048567	0.016813	0.059929	0.022678	0.044701	0.05638
roc_auc_ovr_macro_mean	0.98415	0.983052	0.988979	0.980733	0.968972	0.977948	0.982208	0.97712	0.924949	0.923331
roc_auc_ovr_macro_sd	0.004836	0.006965	0.012893	0.008022	0.009728	0.011295	0.008382	0.004555	0.008003	0.019441
average_precision_macro_mean	0.901152	0.862601	0.908229	0.858437	0.817372	0.850144	0.863179	0.861603	0.736444	0.700623
average_precision_macro_sd	0.019101	0.038714	0.054724	0.04877	0.031539	0.042019	0.058946	0.030892	0.027465	0.090274
epochs_run_mean	52.6	54.6	76.2	85.4	58.4	65.6	73.6	65.6	67.6	61.8
runtime_sec_mean	256.8073	272.601	381.169	420.7808	295.6861	323.5275	368.155	330.5826	340.6902	323.135
runtime_sec_sd	70.30898	29.85758	24.3335	107.72	100.4152	84.28933	66.19519	108.7951	140.7058	99.37309

### Model attribution

Random Forest feature attribution showed that predictive contribution was not uniformly distributed across the skeleton. Joint-level and body-region summaries identified concentrated attribution across selected anatomical regions and individual joints, with the full ranked feature set retained for transparent inspection. **Figure 5** presents the body-region and joint-level attribution summaries, while **Figure 6** maps joint-level attribution onto the H36M-17 skeleton. **Table 6** provides Body-region attribution summary from the strongest kinematic baseline.

**Figure 5:**
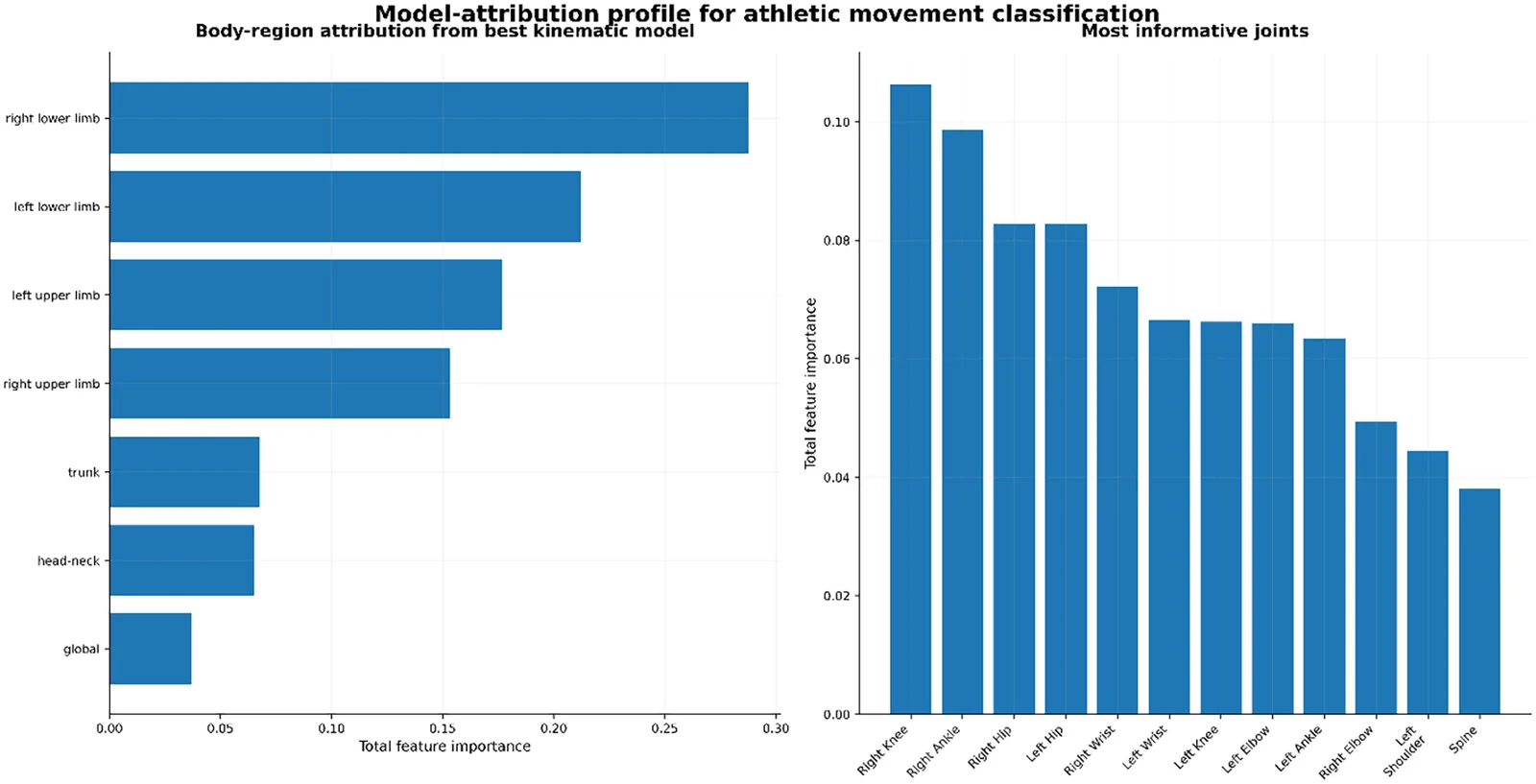
Body-region and joint-level model attribution for athletic movement classification. The figure summarizes Random Forest feature importance aggregated across anatomical body regions and individual H36M-17 joints.

**Figure 6:**
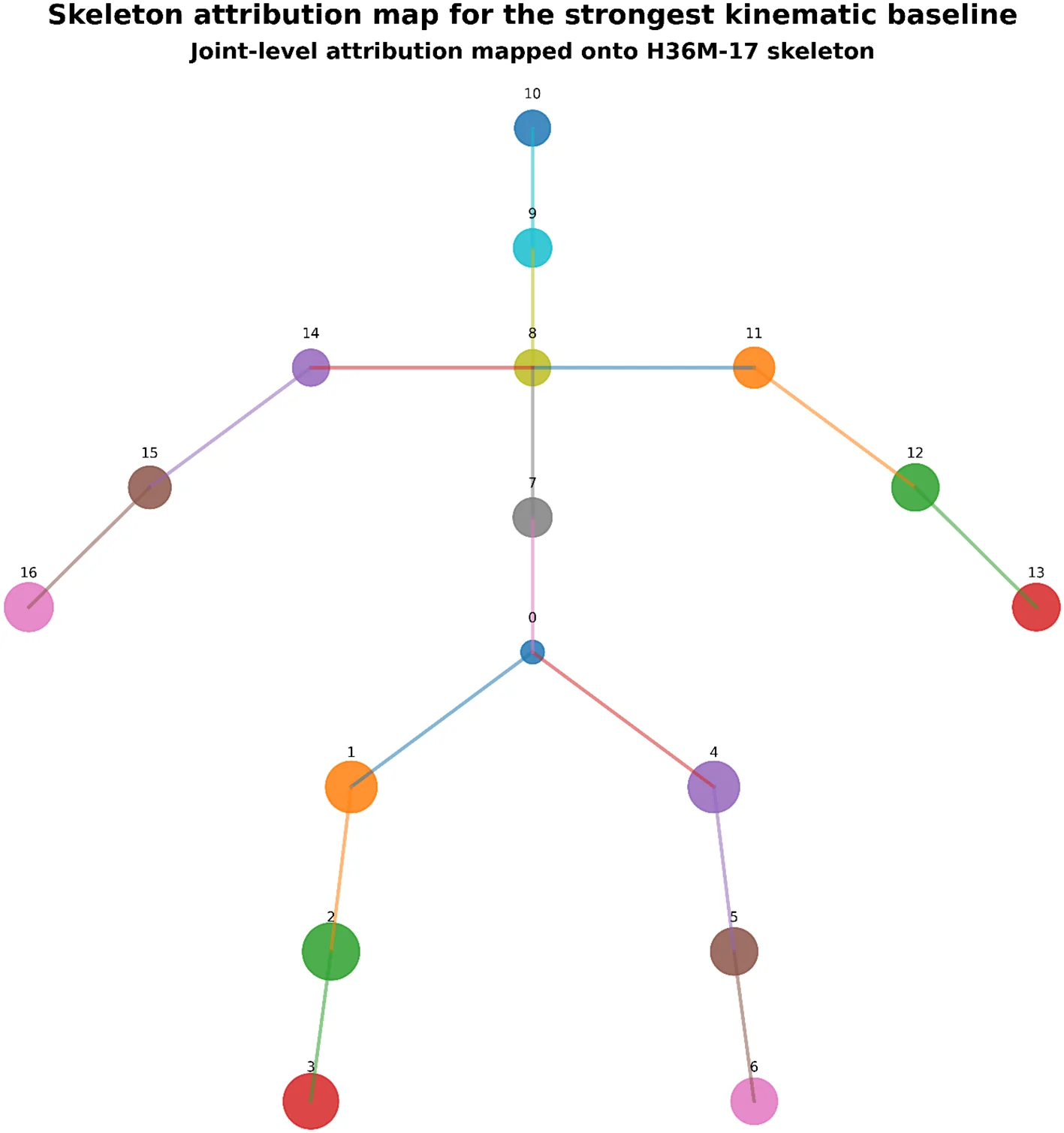
Joint-level attribution mapped onto the H36M-17 skeleton. Node size reflects aggregated model-attribution strength for each joint, showing spatial heterogeneity in the contribution of skeleton regions to athletic movement classification.

**Table 6:** Body-region attribution summary from the strongest kinematic baseline.

Body region	Total importance	Mean importance	Features
right lower limb	0.2877	0.00436	66
left lower limb	0.2122	0.00322	66
left upper limb	0.1769	0.00268	66
right upper limb	0.1534	0.00232	66
trunk	0.0676	0.00102	66
head-neck	0.0650	0.00148	44
global	0.0371	0.00286	13

### Robustness and domain-shift validation

Extended topology evaluation incorporated anatomically informed and motion-based filters alongside stronger tree-based models and a compact adaptive graph model. **Figure 7** shows that the strongest performance was achieved by ExtraTrees-Balanced with identity/no-graph features, exceeding the original Random Forest baseline by approximately 0.052 macro-F1. Motion-based filters also produced competitive results, whereas anatomical and anatomically informed variants remained near the original baseline. Overall, model type and feature representation had a greater effect on performance than topology choice, and the adaptive temporal graph model remained below the leading kinematic approaches, reaching approximately 0.688 macro-F1.

**Figure 7:**
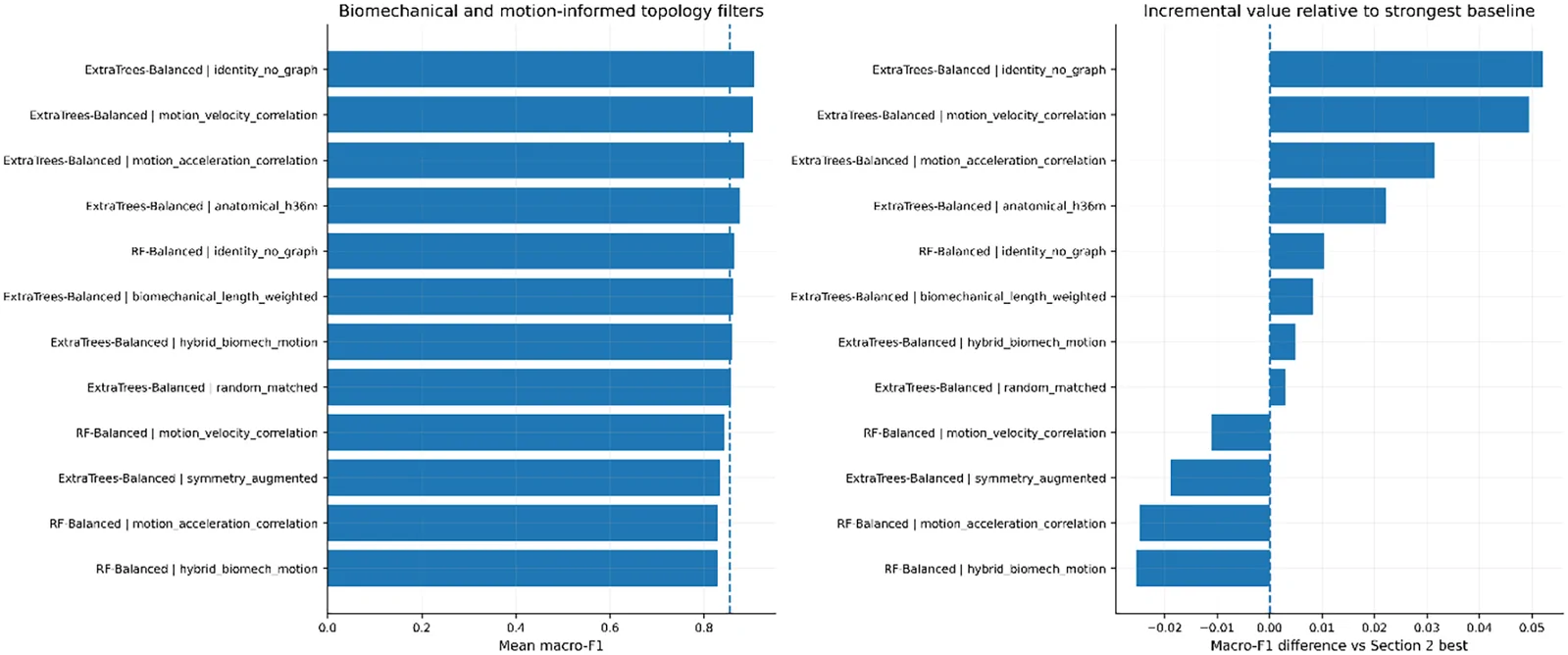
Topology evaluation across identity, anatomical, anatomically informed, motion-based, hybrid, random, and dense graph representations, with model comparisons on the left and macro-F1 differences relative to the baseline on the right.

Pose-perturbation tests were conducted to evaluate downstream sensitivity to imperfect skeleton inputs. **Figure 8** shows that the classifier was robust to mild coordinate noise and moderate frame loss, but highly sensitive to severe noise and missing joints. Clean performance was approximately 0.873 macro-F1; mild noise had minimal effect at approximately 0.871; frame loss reduced performance to approximately 0.829 at 10% and 0.801 at 25%; stronger noise caused larger degradation to approximately 0.693 and 0.361; and joint masking caused near-complete failure at approximately 0.003. These results confirm that the system should be interpreted as a downstream pose-derived movement classifier, not as a full video-based, clinically deployable, or injury-risk prediction pipeline.

**Figure 8:**
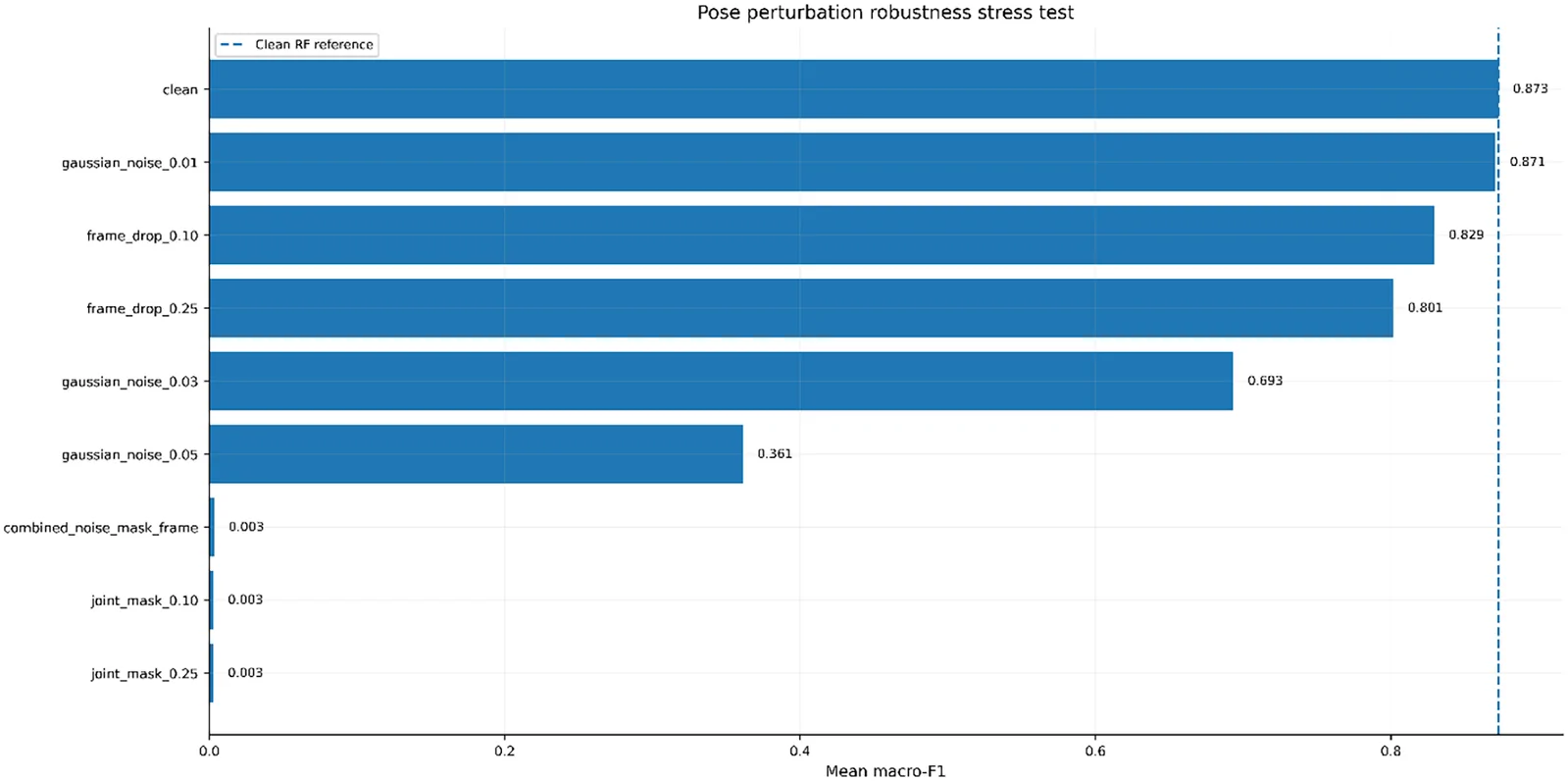
Robustness under noise, frame loss, and joint masking, showing stable performance under mild noise but collapse with missing joints.

Subject-domain-shift analysis showed uneven subject-action coverage and subject-dependent holdout performance. Because S4 had insufficient common-label overlap, complete multi-class subject-holdout evaluation could not be performed for that subject. In the completed folds, macro-F1 values were 0.600 for S1, 0.895 for S2, 0.938 for S3, and 0.874 for S5. These results highlight subject-action fragmentation and limited cross-subject robustness. Therefore, the subject-domain-shift findings should be interpreted as a sensitivity analysis rather than as evidence of broad subject-level generalizability.

Subject-domain-shift analysis showed limited and subject-dependent generalization, as shown in **Figure 9**. Uneven label distribution across subjects prevented full leave-one-subject-out evaluation and excluded S4 from complete multi-class testing. In the completed folds, macro-F1 values were 0.600 for S1, 0.895 for S2, 0.938 for S3, and 0.874 for S5. These results highlight subject-action fragmentation and limited cross-subject robustness rather than broad subject-level generalizability.

**Figure 9:**
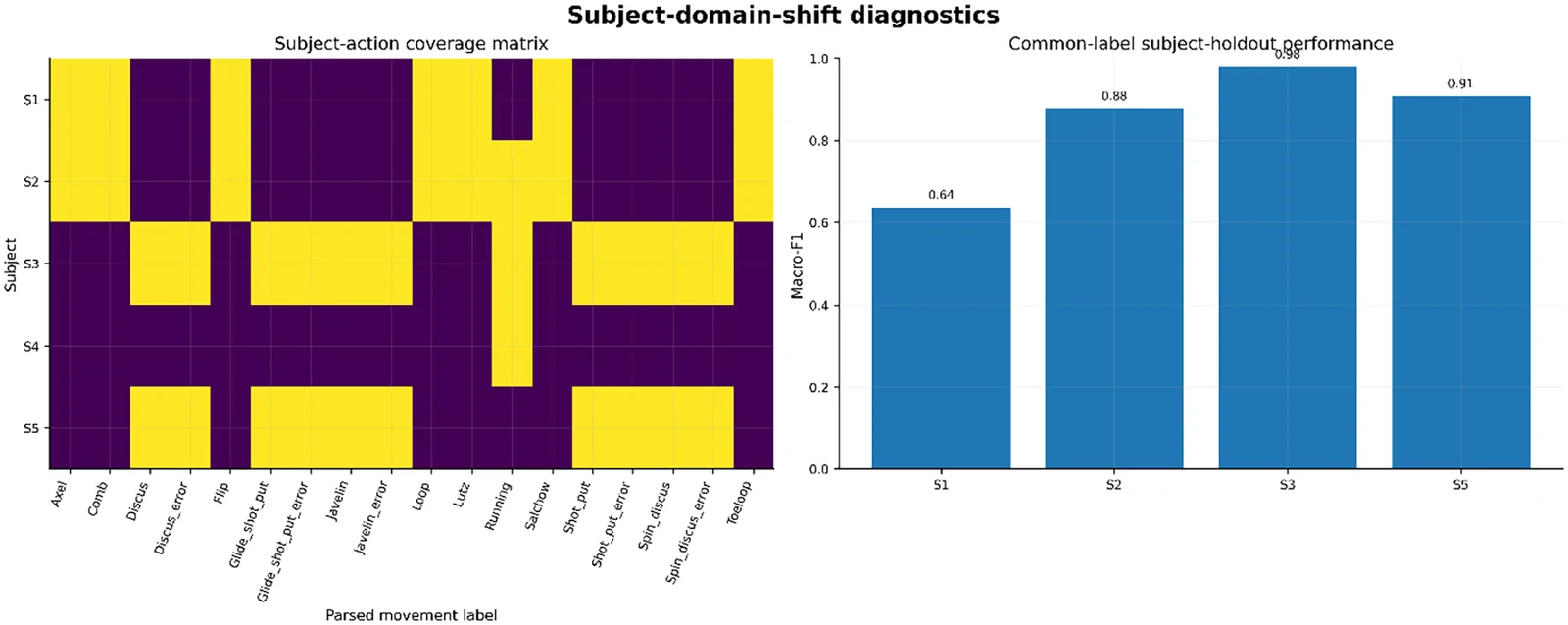
Subject-label coverage and holdout performance, illustrating uneven label distribution and subject-dependent accuracy.

## Discussion

### Principal findings

This study evaluated normalized 3D skeleton kinematics and topology-sensitive skeleton-graph representations for athletic movement classification using AthletePose3D. The main finding was that a carefully constructed kinematic benchmark based on root-centered and scale-normalized H36M-17 pose summaries produced the strongest predictive performance, with the class-balanced Random Forest achieving a mean macro-F1 of 0.854 and balanced accuracy of 0.844 across repeated seeds. This result indicates that compact sequence-level descriptors of joint position, range, velocity, acceleration, motion energy, and body-region structure contain substantial discriminative information for athletic movement labels. At the same time, the deep graph and topology-filter analyses showed that graph structure remained scientifically relevant, but not because graph models outperformed the strongest classical baseline. Instead, topology selection materially affected skeleton-based performance, and arbitrary dense topology degraded performance. Therefore, the central interpretation is that AthletePose3D supports strong 3D kinematic movement classification and topology-aware validation, while graph learning should be framed as a sensitivity and interpretability layer rather than as an automatically superior modeling strategy.

### Physiological relevance to human movement

The kinematic representations evaluated in this study describe observable spatial and temporal characteristics of human movement. Joint displacement and range characterize the spatial organization of movement, while velocity and acceleration describe temporal changes in segmental motion. Together with motion-energy and inter-joint distance features, these descriptors provide quantitative representations of movement organization and multi-segment coordination that are relevant to the study of human motor performance (Mathis et al., 2018; Karashchuk et al., 2021; Avogaro et al., 2023; Cotton et al., 2023; Kakavand et al., 2024; Panconi et al., 2024). Athletic actions involve coordinated contributions from multiple body segments, and differences among running, jumping, throwing, kicking, and related technique variants can therefore be reflected in the spatial and temporal organization of skeletal motion (Ingwersen et al., 2023; Suzuki et al., 2025). The present results show that these interpretable kinematic characteristics contain sufficient information to distinguish movement classes and, within the present dataset, preserve more classification-relevant information than the evaluated graph-based representations.

The regional attribution findings provide an additional movement-level perspective. Greater model attribution in the lower limbs and selected upper-limb regions indicates that the classifier relied disproportionately on movement information arising from these segments. Such a pattern is compatible with the multi-segment nature of the athletic actions represented in AthletePose3D, in which lower-limb movement and support occur together with action-specific trunk and upper-limb motion (Ingwersen et al., 2023; Suzuki et al., 2025). However, these findings concern the informational value of pose-derived movement features for classification rather than direct evidence of underlying physiological or biomechanical mechanisms. Markerless pose-derived kinematics can provide useful information about movement while remaining sensitive to pose-estimation accuracy, movement speed, camera geometry, and task context (Avogaro et al., 2023; Kakavand et al., 2024; Panconi et al., 2024). Accordingly, the present findings should not be interpreted as measurements of muscle activation, neuromuscular control, metabolic demand, joint loading, or other physiological processes. The study therefore contributes to human-movement physiology at the level of computational characterization of observable movement dynamics rather than direct physiological inference.

### Sports-pose context

The dataset-level findings are consistent with recent work emphasizing that conventional pose-estimation and action-recognition datasets often underrepresent high-speed, high-acceleration sports motion. AthletePose3D was introduced specifically to address this gap by providing athletic movements with approximately 1.3 million frames and 165,000 postures across 12 broader sports-motion types (Yeung et al., 2025). SportsPose similarly argued that sports movements are more dynamic than common benchmark motions and showed that sport-specific 3D pose datasets are needed for valid evaluation of athletic movement (Ingwersen et al., 2023). AthleticsPose further reinforced this point by showing that models trained on authentic sports motion transfer better than models trained on imitated sports motion, while also noting persistent difficulty for high-speed metrics such as velocity estimation (Suzuki et al., 2025). In this context, the present analysis contributes a complementary classification-oriented evaluation: rather than estimating pose from images, it tested whether the available 3D skeleton sequences and derived kinematic features could support robust athletic movement discrimination. The use of 18 filename-parsed labels in this study should be interpreted as a modeling-level label set that includes technique and error variants, not as a contradiction of the dataset-level description of 12 broader sports-motion categories.

### Kinematic baseline

The strongest original baseline performance was obtained by the class-balanced Random Forest, which exceeded logistic regression, multilayer perceptron, and gradient boosting baselines on macro-F1 and balanced accuracy. This result is plausible because the engineered kinematic features summarize both spatial body configuration and temporal movement variation, while Random Forests can model nonlinear interactions among features without requiring the larger sample sizes typically needed by deep neural architectures. Random Forests are also well suited to tabular, medium-scale, heterogeneous feature spaces and provide built-in feature-importance estimates that can be aggregated for model-attribution analysis (Breiman, 2001; Pedregosa et al., 2011). The high macro ROC-AUC and average precision values suggest that the baseline separated many movement classes effectively; however, the macro-F1 and class-wise results remain more informative than ROC-AUC alone because several action/error labels had limited test support. Compared with large-scale action-recognition benchmarks such as Kinetics and NTU RGB+D 120, the present dataset is much smaller in subject and class coverage, so the absolute score should not be interpreted as directly comparable to large benchmark accuracies (Liu et al., 2019). Instead, the result establishes a strong within-dataset benchmark for subsequent graph and topology analyses.

### Graph topology

The graph results show that topology matters, but they do not support a claim that graph models universally outperform strong kinematic baselines. The best deep graph configuration was the GCN with anatomical H36M topology, achieving a mean macro-F1 of 0.752 and balanced accuracy of 0.774. This was lower than the Random Forest baseline but stronger than poorly structured dense graph controls. This pattern aligns with the skeleton-action literature, where ST-GCN established the value of representing the human skeleton as a spatial-temporal graph, but later studies showed that the definition and adaptation of topology are central to performance (Yan et al., 2018; Chen Y, et al., 2021). Recent topology-focused models, including CTR-GCN, HD-GCN, BlockGCN, and topology-symmetry-enhanced GCNs, have emphasized that skeletal adjacency should not be treated as a trivial fixed design choice, because joint connectivity, symmetry, long-range dependencies, and action-specific joint relations can all affect representation learning (Chen Y, et al., 2021; Lee et al., 2022; Zhou et al., 2023; Liang et al., 2024). The present findings are consistent with that literature: graph construction was not neutral, fully connected topology was harmful, and anatomical structure provided a more meaningful control than arbitrary dense aggregation.

### Fast topology-filter analysis

The fast graph-filter sensitivity analysis examined whether topology-induced smoothing improved the strongest kinematic representation. The identity/no-graph Random Forest configuration was closest to the normalized kinematic baseline, with macro-F1 of approximately 0.852 and a difference of −0.002 relative to the strongest baseline. Anatomical, symmetry-augmented, random-matched, and distance-based graph filters all reduced performance to varying degrees, and fully connected filtering produced the largest degradation. This negative result is important because it prevents overinterpretation of graph filtering as automatically beneficial. It also supports the argument that movement-classification signal in AthletePose3D is strongly preserved in the raw normalized kinematic summaries, and that indiscriminate smoothing across joints can suppress discriminative joint-specific information. This interpretation is compatible with recent skeleton-recognition studies showing that both local skeletal structure and distant or higher-order joint relationships may be informative, but that inappropriate topology design can limit performance (Chen Z, et al., 2021; Zhou et al., 2022; Zhou et al., 2023). The fast topology-filter analysis should therefore be reported as sensitivity evidence, not as evidence of graph-model superiority.

### Validation

The validation results strengthen the credibility of the pipeline because performance was assessed through multiple complementary checks rather than a single model score. The official train/validation/test split was preserved, repeated-seed evaluation was used for baseline and graph models, topology sensitivity was tested using several graph controls, and subject-holdout sensitivity was added as an additional generalization check. The subject-holdout analysis showed subject-dependent generalization: S2, S3, and S5 produced strong macro-F1 values, whereas S1 was weaker and S4 was skipped because only one common class was available. This pattern is important because sports movement data can vary substantially across athletes, technique variants, camera settings, and sequence composition. Recent sports-pose work has similarly reported sensitivity to real-world factors such as camera view, subject scale, and task-specific motion dynamics (Baumgartner and Klatt, 2023; Suzuki et al., 2025). Therefore, the subject-holdout findings should be interpreted cautiously. They support generalization sensitivity rather than definitive cross-subject robustness across all athletes and action labels.

### Model attribution

The model-attribution results showed that the Random Forest baseline relied unevenly on body regions and joints, with higher aggregated importance in lower-limb regions and selected upper-limb joints. This is plausible for a dataset containing athletic movements such as running, jumping, throwing, kicking, skating, discus, javelin, and shot-put variants, where lower-limb support, trunk transfer, and upper-limb action segments can all contribute to movement discrimination. However, feature importance from tree ensembles should be interpreted as model attribution rather than causal biomechanical evidence. Random Forest importance indicates which input features were useful to the trained classifier under the present preprocessing and label structure; it does not prove that a joint or body region is biomechanically causal for a movement class. This distinction is especially important because recent markerless and sports-pose studies emphasize that pose-derived kinematics can be informative but remain sensitive to estimation accuracy, movement speed, camera geometry, and task context (Avogaro et al., 2023; Kakavand et al., 2024; Panconi et al., 2024). The attribution results are therefore best used to improve transparency and generate biomechanical hypotheses for future validation, not to make clinical or injury-risk claims.

### Relation to modern skeleton recognition

The present findings also help position classical kinematic baselines relative to modern skeleton-learning models. Contemporary skeleton-action recognition has progressed from fixed spatial-temporal graph convolution to adaptive topology refinement, hierarchical graph decomposition, hypergraph attention, and skeletal-temporal transformer designs (Yan et al., 2018; Chen Y, et al., 2021; Lee et al., 2022; Zhu et al., 2022; Do and Kim, 2024). These models often achieve strong performance on large benchmark datasets, where thousands of samples and many subjects support deep representation learning. In contrast, the present AthletePose3D classification setting used a smaller camera-1 sequence subset with 18 parsed labels and several low-support subclasses. Under these conditions, a strong tabular kinematic representation paired with a class-balanced Random Forest was more effective than the implemented GCN/GAT models. This does not undermine graph learning as a field; rather, it shows that deep graph models should be benchmarked against strong classical baselines and evaluated with topology controls before performance claims are made. Motion representation studies such as MotionBERT also suggest that large-scale pretraining and richer temporal representation learning can improve transfer across human-motion tasks, indicating that future work may benefit from pretrained motion encoders rather than training small graph models from scratch (Zhu et al., 2022).

### Robustness, topology, and domain-shift implications

The analyses clarify the scientific contribution of the study. Rather than demonstrating that graph models outperform strong kinematic baselines, the results show that AthletePose3D movement classification is best understood as a robustness-aware and topology-aware benchmarking problem. The topology experiments extend beyond comparisons with random or dense controls by incorporating anatomically informed bone-length-weighted, motion-velocity-correlation, motion-acceleration-correlation, and hybrid anatomy-motion graph filters. Despite these enhancements, the strongest performance is achieved by tree-based kinematic models, particularly ExtraTrees-Balanced, while the compact adaptive temporal graph model remains below the leading kinematic baselines. This outcome supports a more cautious interpretation: topology design is scientifically meaningful because it alters representation behavior and reveals the limits of graph assumptions, but it does not guarantee superior classification in a small, fragmented sports-pose subset.

The perturbation analysis further refines the scope of the study by showing that the pipeline is robust to mild coordinate noise and moderate frame loss but fails under missing-joint perturbations. This indicates that practical deployment would require reliable upstream pose extraction and effective joint-confidence handling before downstream classification. In addition, the subject-domain-shift analysis highlights a key dataset constraint: uneven subject-action coverage limits broad cross-subject generalization and prevents complete leave-one-subject-out evaluation for all subjects. Taken together, these findings position the work as a transparent computational validation benchmark for athletic movement classification, emphasizing strong kinematic baselines, behavior-informed topology evaluation, perturbation robustness, and subject-domain-shift reporting rather than universal graph-model superiority.

### Limitations

This study has several limitations. First, it relies on curated H36M 3D pose arrays from AthletePose3D and therefore represents a downstream pose-based classification task rather than an end-to-end raw-video pipeline. It does not directly address real-world pose-extraction challenges such as occlusion, motion blur, camera-view variation, or multi-athlete scenes, and the perturbation tests should be interpreted only as controlled approximations of imperfect skeleton inputs. Second, subject-action fragmentation limited uniform label distribution, prevented complete leave-one-subject-out evaluation for all subjects, and constrained subject-level generalizability claims. Third, graph-based models did not outperform tree-based kinematic baselines, indicating that topology design is informative for validation but not automatically superior for classification in this dataset. Fourth, the strongest results depended on engineered sequence-level features that summarize temporal dynamics and may encode dataset-specific patterns. Fifth, several movement classes had limited support, reducing the stability of class-wise metrics. Finally, feature-attribution results should be interpreted only as model-level explanations, not as causal biomechanical evidence, physiological mechanisms, injury-risk indicators, or clinical/performance recommendations.

### Implications

The results have three main implications. First, sports-pose datasets such as AthletePose3D can support movement-classification research beyond pose-estimation benchmarking, provided that label structure, splits, subject coverage, and camera-view design are audited carefully. Second, strong kinematic baselines should be included in skeleton-learning studies because interpretable sequence-level features may outperform deeper models when sample size is limited and labels are heterogeneous. Third, topology sensitivity should be treated as an essential validation component in skeleton-graph modeling. The poor performance of fully connected controls and the stronger performance of anatomical topology indicate that the graph structure is not merely an implementation detail. This supports a more cautious and reproducible framework for athletic movement modeling: establish dataset readiness, benchmark interpretable kinematic features, test graph/topology sensitivity, report subject-level validation, and present attribution as model explanation rather than biomechanical causality.

## Conclusion

In summary, normalized 3D skeletal kinematics provided a strong and interpretable representation of athletic movement in AthletePose3D. Sequence-level characteristics of joint position, movement range, velocity, acceleration, motion magnitude, and body-region relationships retained substantial information for distinguishing athletic movement classes, and the leading tree-based kinematic models consistently outperformed the evaluated fixed and adaptive graph representations. Topology nevertheless influenced model behavior, demonstrating that anatomical structure remains an important consideration when validating skeleton-based movement representations. Model-attribution findings further showed that classification-relevant information was distributed unevenly across body regions, with prominent contributions from the lower limbs and selected upper-limb features. From a human-movement physiology perspective, these findings indicate that pose-derived kinematic descriptors can provide an interpretable computational representation of the spatial and temporal organization of athletic movement. They should not, however, be interpreted as direct measurements of physiological processes, causal biomechanical mechanisms, injury risk, or clinical outcomes. The study therefore supports topology-aware and robustness-aware analysis of 3D skeletal kinematics as a tool for computational investigation of athletic movement dynamics.

## Data Availability

The original contributions presented in the study are included in the article/supplementary material. Further inquiries can be directed to the corresponding author.
